# New Appliances & Things Medical

**Published:** 1910-04-02

**Authors:** 


### NEW APPLIANCES & THINGS MEDICAL.

[We shall be glad to receive at our Office, 28 & 29 Southampton Street, Strand, London, W.C., from the manufacturers, specimens of all new preparations and appliances.]

### THE SYNTAX SYSTEM OF FILING.

(E. Norman and Co., expert classifiers and routine specialists, 26 Great St. George's Street, Leeds. Telegrams: Method, Leeds.)

The Syntax system of filing is one that should commend itself to hospital workers. It is a grave question this, the proper system of filing which institutions should adopt to deal with the immense mass of correspondence that accumulates in the course of a few years in the best regulated hospitals. Every institutional worker will doubtless have his own favourite methods. Cabinet methods, card methods, index methods, colour scheme methods, and the many other contrivances that the ingenuity of classifiers has invented will suggest themselves, and he who wishes to secure a perfect and reliable system must try them all and see which one gives the best results with the greatest economy of time and the least possible trouble and worry. To anyone in search of a perfect system we can recommend a trial of the Syntax. It is, to begin with, a natural system, with a clearly defined starting-point, and its application is perfectly general. It is suitable, therefore, not only for the secretarial department, but for the dozen and one other departments which are to be found in connection with a modern up-to-date hospital, and we think it will be found specially convenient for use in the out-patient department, where the present systems are all more or less failures. Anyone who wishes to test the usefulness and general applicableness of the system for himself should write for the informative booklet which the agents supply on application. This gives full details of the method, and if the information it contains is not sufficient to show the merits of the method, the agents will be glad to give a personal demonstration of the adaptability of the system to the various departments of a hospital.

